# Localisation and Function of the Endocannabinoid System in the Human Ovary

**DOI:** 10.1371/journal.pone.0004579

**Published:** 2009-02-24

**Authors:** Mona R. El-Talatini, Anthony H. Taylor, Janine C. Elson, Laurence Brown, Allan C. Davidson, Justin C. Konje

**Affiliations:** 1 Endocannabinoid Research Group, Reproductive Sciences Section, Department of Cancer Studies & Molecular Medicine, University of Leicester, Leicester, United Kingdom; 2 Assisted Conception Unit, Leicester Royal Infirmary, University Hospitals of Leicester NHS Trust, Leicester, United Kingdom; 3 Department of Pathology, Leicester Royal Infirmary, University Hospitals of Leicester NHS Trust, Leicester, United Kingdom; Cincinnati Children's Research Foundation, United States of America

## Abstract

**Background:**

Although anandamide (AEA) had been measured in human follicular fluid and is suggested to play a role in ovarian follicle and oocyte maturity, its exact source and role in the human ovary remains unclear.

**Methods and Findings:**

Immunohistochemical examination of normal human ovaries indicated that the endocannabinoid system was present and widely expressed in the ovarian medulla and cortex with more intense cannabinoid receptor 2 (CB2) than CB1 immunoreactivity in the granulosa cells of primordial, primary, secondary, tertiary follicles, corpus luteum and corpus albicans. The enzymes, fatty acid amide hydrolase (FAAH) and *N*-acyclphosphatidylethanolamine-phospholipase D (NAPE-PLD), were only found in growing secondary and tertiary follicles and corpora lutea and albicantes. The follicular fluid (FF) AEA concentrations of 260 FF samples, taken from 37 infertile women undergoing controlled ovarian hyperstimulation for in vitro fertilisation and intracytoplasmic sperm injection with embryo transfer, were correlated with ovarian follicle size (P = 0.03). Significantly higher FF AEA concentrations were also observed in mature follicles (1.43±0.04 nM; mean±SEM) compared to immature follicles (1.26±0.06 nM), P = 0.0142 and from follicles containing morphologically assessed mature oocytes (1.56±0.11 nM) compared to that containing immature oocytes (0.99±0.09 nM), P = 0.0011. ROC analysis indicated that a FF AEA level of 1.09 nM could discriminate between mature and immature oocytes with 72.2% sensitivity and 77.14% specificity, whilst plasma AEA levels and FF AEA levels on oocyte retrieval day were not significantly different (P = 0.23).

**Conclusions:**

These data suggest that AEA is produced in the ovary, is under hormonal control and plays a role in folliculogenesis, preovulatory follicle maturation, oocyte maturity and ovulation.

## Introduction

Endocannabinoids (endogenous cannabinoids) are unsaturated fatty acid derivatives widely distributed in the human body [1]. Anandamide (AEA), the most studied endocannabinoid, mimics some of the central and peripheral effects of Δ^9^ - tetrahydrocannabinol (Δ^9^-THC), the psychoactive component of marijuana [2]. AEA, an ethanolamine amide of arachidonic acid, the precursor of prostaglandins and leukotrienes [3] is synthesised on demand through the sequential actions of *N*-acyltransferase (NAT) and a molecule-specific enzyme *N*-acyclphosphatidylethanolamine-phospholipase D (NAPE-PLD) [4]. AEA binds to and activates cannabinoid receptors of which there are two main isoforms, cannabinoid receptor 1 (CB1) and CB2 [5]. In the human, CB1 is considered to be the main receptor in the central nervous system [6] and is expressed in many other peripheral tissues such as the adrenal gland, ovaries, uterus, testis, prostate [5], and placenta [7]. In contrast, CB2, which is not expressed in the brain except in conditions of extreme stress, is mainly found in immune-based tissues [8] such as the spleen, tonsils, thymus, bone marrow, B-cells, natural killer cells, monocytes, polymorphic mononuclear cells, neutrophils and T8- and T4-postive cells [5] and more recently in the 1^st^ trimester trophoblast [9]. On binding and internalisation, AEA is degraded to arachidonic acid and ethanolamine by the microsomal enzyme fatty acid amide hydrolase (FAAH) [10]. FAAH and NAPE-PLD are considered to be the main regulators of AEA in target tissues often referred to as ‘anandamide tone’ [11]. The ligands (endocannabinoids), receptors and enzymes as a group constitute the main components of the endocannabinoid system.

Our knowledge about the effect of cannabinoids on the ovary comes from studies in animals [12] and marijuana users [13]. Studies in the *in-vitro* rat ovary model demonstrated that Δ^9^-THC exerts a direct inhibitory effect on folliculogenesis [12] and ovulation [14] whereas it causes anovulation in rats [15], rabbits and rhesus monkeys [16] as a result of LH surge disruption [17]. The effects of cannabis and THC on the human ovary have been studied and shown to be inconsistent [18] mainly due to the development of tolerance among chronic users [18] and the timing of administration of cannabis in relation to the phases of the menstrual cycle [13]. While in some studies, chronic cannabis smokers appeared to have normal menses after intensive smoking [13], other studies showed increased anovulatory cycles and a short luteal phase. Nevertheless, a direct adverse effect on the ovary were clearly observed as cannabis users were at a higher risk of primary infertility due to anovulation [19], and even when these women had IVF treatment, they produced poor quality oocytes and lower pregnancy rates compared to non-users [20].

AEA has been demonstrated in ovarian follicular fluids at the time of oocyte retrieval in IVF cycles suggesting that it may play a role in ovarian follicle or oocyte maturity [21], [22]. However, the source of AEA in the follicular fluid and its possible role within the ovary remains poorly understood. Therefore, our study aimed to localise the endocannabinoid system in the ovary and to investigate whether follicular fluid or plasma AEA levels are related to physiologically important ovarian events such as folliculogenesis, the size and maturity of preovulatory follicle, oocyte maturity, and ovulation.

## Materials and Methods

Each volunteer signed an informed written consent prior to entry in to the study which was approved by the Leicestershire and Rutland Research Ethics Committee.

Our study was in 2 parts; the first was mainly to localise the endocannabinoid system in the ovary using immunohistochemistry, and the second to investigate the role of AEA in ovarian follicles in relation to folliculogenesis, follicle size and oocyte maturity.

### Subjects

For the immunohistochemical studies, 12 ovarian tissue blocks were collected prospectively from women with regular (cycle length 28–32 days) menstrual cycles having a hysterectomy and bilateral salpingo-oophorectomy for benign pathology such as; heavy periods, benign ovarian cyst or prophylactic oophorectomy for family history of ovarian cancer. The woman who had a family history of ovarian cancer was not a carrier of the BRCA1 gene. None of the volunteers had been on any medication for at least one month prior to surgery. The ovaries were confirmed by a gynaecological pathologist to be normal. Control tissues including fetal membranes (for CB1, CB2 and FAAH) and secretory phase endometrium (for NAPE-PLD) were obtained from women undergoing elective Caesarean section at term [9] and hysterectomy for benign conditions such as myoma or dysfunctional uterine bleeding [23], respectively. All tissues were fixed in 10% neutral buffered formalin for 4 days before being embedded in paraffin wax.

For the assessment of follicular fluid AEA concentrations and the possible role of AEA in ovarian physiology, a total of 37 women undergoing ovarian stimulation for *in vitro* fertilisation (IVF) and intracytoplasmic sperm injection (ICSI) with embryo transfer (ET) between July 2007 and December 2007 were recruited into the study at the Assisted Conception Unit of the Leicester Royal Infirmary Hospital. All women were healthy and had no other medical disorders, had not used cannabis in the last 10 years and had a basal FSH of ≤10 IU/l in the period prior to starting IVF/ICSI-ET. Eight percent of the volunteers smoked the remainder did not.

### Controlled ovarian stimulation protocol, follicular fluid sampling and oocyte retrieval

Ovarian stimulation was performed using a long protocol, with pituitary down-regulation with the gonodatrophin releasing hormone (GnRH) agonist Supercur (Aventis Pharma Ltd, Kent, UK) commenced in the mid luteal phase of the previous cycle and continued until ovulatory human chorionic gonadotrophin (hCG) was given [24]. Stimulation was initiated with either human menopausal gonadotrophin (hMG) Menopur (Ferring, Langley, UK) or recombinant follicle stimulating hormone (rFSH) Puregon (Organon Laboratories Ltd, Cambridge, UK) or a combination of rFSH and hMG [25] once there was no sonographic evidence of ovarian follicular activity and serum estradiol levels were below 200 pmol/L. The dosage was based on the patient's age, BMI and early follicular phase serum FSH levels [26]. Follicular maturation was assessed by serial (every 2–3 days) transvaginal ultrasound scan and serum estradiol measurements [27]. hCG 10,000 IU (Pregnyl; Organon Laboratories Ltd, Cambridge, UK) was administered subcutaneously to induce final oocyte maturation when at least 4 follicles measuring at least 17 mm in diameter combined with an endometrial thickness of 8 mm were observed. Thirty-six hours after hCG administration, oocyte retrieval was performed transvaginally under ultrasound guidance and intravenous sedation with a combination of Propofol (Propoven 1%; Fresenius Kabi Ltd, Cheshire, UK), Alfentanil hydrochloride (Rapifen; Janssen-Cilag Ltd, Buckinghamshire, UK) and Midazolam (Hypnovel; Roche, Hertfordshire, UK). All the identified follicles were aspirated until the follicle wall collapsed. Prior to the administration of sedation, 4 ml of blood was collected into an EDTA tube for AEA assay. The aspirated fluid was then examined under a low power stereomicroscope (Nikon SM200) by an experienced embryologist for the presence of oocytes. The corona-cumulus-oocyte complex was identified, and removed with a Pasteur glass pipette (Poulten & Graf Ltd, Barking, UK). The follicular fluid from each follicle was thereafter collected into Kimble Scintillation vials for AEA measurements.

### Measurement of Plasma and follicular fluid AEA concentrations

Blood and follicular fluid samples were processed within two hours of collection. Plasma and follicular fluid AEA concentrations were quantified using the Ultra Performance Liquid Chromatography-Mass Spectrometry (UPLC-MS/MS) method which we previously described [28]. AEA measurements were only made form samples that were at least 1 ml in volume as our extraction method was not valid for lower volumes.

### Assessment of ovarian follicle size, oocyte maturity and embryo quality

The follicle size was determined by measuring the volume of the fluid aspirated [29] obtained from individual follicles with Falcon plastic serological sterile pipettes (Becton Dickinson UK Ltd, Plymouth, UK). Standard techniques for ICSI and *In -vitro* insemination and culture were followed [30]. Only the oocytes from patients having ICSI were assessed for maturity since oocytes from IVF are not routinely assessed for maturity. Oocytes were mechanically cleaned from their surrounding cumulus cells by aspiration through a plastic Stripper Tip (∼125 µl inner diameter; MidAtlantic Diagnostics, Berlin, Germany). All the oocytes were examined by an embryologist with an inverted microscope (Olympus I ×70) at a magnification of ×200 and those with a polar body were selected for micromanipulation. Oocyte maturity was based on morphological assessment [31] where (a) germinal vesicle (GV) stage represented very immature oocyte with a germinal vesicle present, (b) metaphase I (MI) stage oocytes were still immature but the germinal vesicle had disappeared and the first polar body had not been extruded and (c) metaphase II (MII) oocytes were mature and the first polar body was present.

Embryo quality was assessed during the second and third day [32] of culture and was defined by the number, shape and size of blastomeres, presence of vacuoles and the grade of fragmentation. The scale was from 1 to 4, where 1 is the best and 4 the poorest quality. Grade 1 embryos have blastomeres which are even and spherical in shape and fill the volume of zona and there is no fragmentation; Grade 2 embryos have blastomeres which are slightly uneven or irregularly shaped and there is up to 10% fragmentation; Grade 3 embryos exhibit fragmentation of not more than 50% of the blastomeres, and grade 4 embryos have gross fragmentation of more than 50% of the blastomeres.

### Immunolocalisation of CB_1_, CB_2_ and FAAH

The CB1 and CB2 rabbit polyclonal antibodies (Sigma-Aldrich Ltd., Poole Dorset, UK) were used at 1∶500 and 1∶250 dilutions, respectively with rabbit IgG (Dako, Glostrup, Denmark) diluted to the same concentration as the negative control. The FAAH rabbit immunised serum (Alpha Diagnostics International, San Antonio, TX) was used at a 1∶2000 dilution with normal rabbit serum (DAKO) diluted to the same concentration as control.

Tissues sections (5 µm) were mounted onto silanized glass microscope slides and dried for 7 days at 37°C prior to use. Slides were de-waxed in xylene three times for 3 min and re-hydrated in graded alcohol for 3 min followed by incubation in distilled water for 3 min. Microwave antigen retrieval was performed for CB1 and CB2 only by incubating the slides in 10 mM citric acid buffer (pH 6.0) heated at 700 watts for 10 min [33]. Endogenous peroxidase activity was then blocked by incubation in 6% H_2_O_2_ in water for 10 min. Blocking of non-specific protein binding sites was performed by incubation in 10% normal goat serum for 10 min at room temperature. Endogenous avidin and biotin sites were blocked using the Avidin-Biotin Blocking Kit (Vector Laboratories) as recommended by the manufacturer. Primary antibodies diluted in tris-buffered saline (TBS; 0.5 M Trizma, 1.5 M NaCl, 2 mM MgCl_2_, pH 7.6; 100 µl/slide) were added and the slides were incubated in a humid chamber overnight at 4°C. Slides were then washed in TBA [Tris-buffered saline containing 0.1% bovine serum albumin (Fraction V; Sigma-Aldrich Ltd.)] for 30 min. After washing the slides for 30 min in TBS, biotinylated goat anti-rabbit antibody (Vector Laboratories) diluted to 1∶400 in TBA was applied for 30 min at room temperature. After an additional wash in TBS, and ABC Elite reagent was applied according to the manufacturer's detailed instructions. After additional washing in TBS for 20 min, DAB was added to each slide (100 µl/slide) for 5 min. Slides were then washed in distilled water for 5 min before counterstaining in Mayer's haematoxylin for 15 seconds. After washing in running tap water for 5 min, slides were dehydrated in graded alcohols, cleared in xylene twice for 6 min before mounting with DPX mounting medium (BDH Poole, Dorset).

### Immunolocalisation of NAPE-PLD

NAPE-PLD immunohistochemistry was performed according to the manufacturer's instructions (ABIN110270; Cayman Chemicals, Ann Arbor, MI). Briefly after de-waxing and rehydration to water, endogenous peroxidase activity was blocked by incubation in 3% H_2_O_2_ in ice-cold water for 15 min and non-specific protein binding sites blocked with 5% normal goat serum in TBS (TBS; 0.5 M Trizma, 1.5 M NaCl, pH 7.4; 100 µl/slide) for 30 min at room temperature. Primary antibodies diluted in TBS (1∶200) were added and the slides were incubated in a humid chamber overnight at room temperature. After washing in TBS containing 0.1% Tween 20 (TBS-T; Sigma-Aldrich Ltd.) three times for 5 min with buffer changes, biotinylated goat anti-rabbit antibody (Dako; Glostrup, Denmark) diluted to 1∶400 in TBS was applied for 30 min at room temperature. After an additional wash in TBS-T, ABC Elite reagent (Vector Laboratories) was applied and the slides washed again. Immunoreactivity was visualised with 3,3′-diaminobenzidine for 5 min and lightly counterstained with Mayer's haematoxylin. After washing in running tap water for 5 min, slides were dehydrated in graded alcohols, cleared in xylene twice for 6 min before mounting with DPX mounting medium (BDH Poole, Dorset).

Photomicrographs were taken on an Axioplan transmission microscope equipped with a Sony DXC-151P analogue camera connected to a computer running Axiovision image capture and processing software (Axiovison version 4.4, Carl Zeiss Ltd., Welwyn Garden City, Hertfordshire, UK). Images were captured at 50×, 200× or 400× magnification in the presence of daylight and medium value neutral density filters with the lamp set at 6400K. Image backgrounds were colour corrected to neutral grey with the use of ColorPilot software (version 4.62; www.colorpilot.com). Immunohistochemical staining was subjectively scored as absent (−), present (+), or intense staining (++).

### Statistics

Demographic and cycle characteristic data are expressed as mean±SD (range) or median where appropriate. Follicular fluid and plasma AEA levels are expressed as mean±SEM. Means were compared using the unpaired Student's *t*-test and P<0.05 was considered statistically significant. For correlations between follicle size and follicular fluid AEA concentrations Spearman correlation test was used.

## Results

The mean age of the 12 volunteers from whom ovarian sections were obtained was 41.8±2.8 (range 39–45 years). The indications for removal of the ovaries were menorrhagia (6 women), benign ovarian cyst (5 women) and prophylactic oophorectomy for a family history of ovarian cancer (1 woman).

### Immunohistochemistry

**Figure 1:**
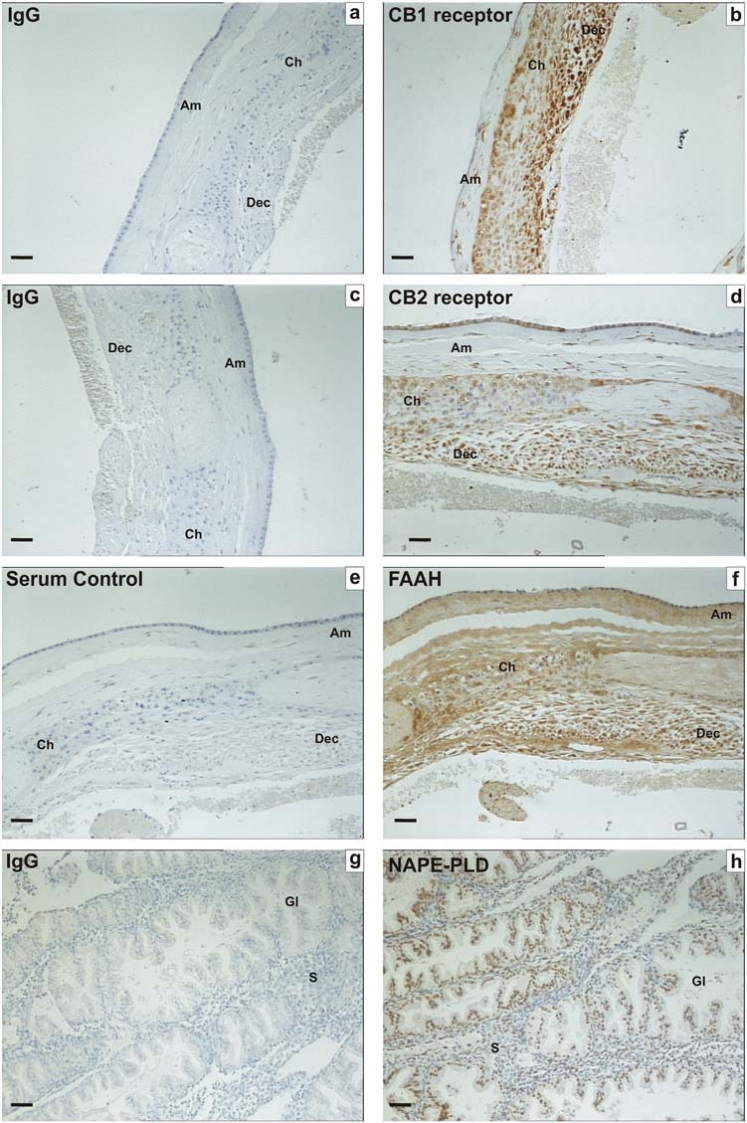
Immunohistochemical staining for CB1, CB2, FAAH and NAPE-PLD on control tissues. Images on the left side of the panel represent the negative control (IgG/serum) and images on the right side of the panel are sections incubated with specific antibodies/antisera. The tissues used in a–f are human fetal membranes whereas g and h are human endometrium. CB1, CB2 and FAAH Immunoreactivity was observed in the amnion (Am), chorion (Ch) and decidua (Dec) of term fetal membranes, whilst NAPE-PLD immunoreactivity was observed mainly in the endometrial glands (Gl) with sparse staining in endometrial stroma (S). The images are representatives from at least six samples and were taken at 200× magnification. Bar = 50 µm.

**Figure 2:**
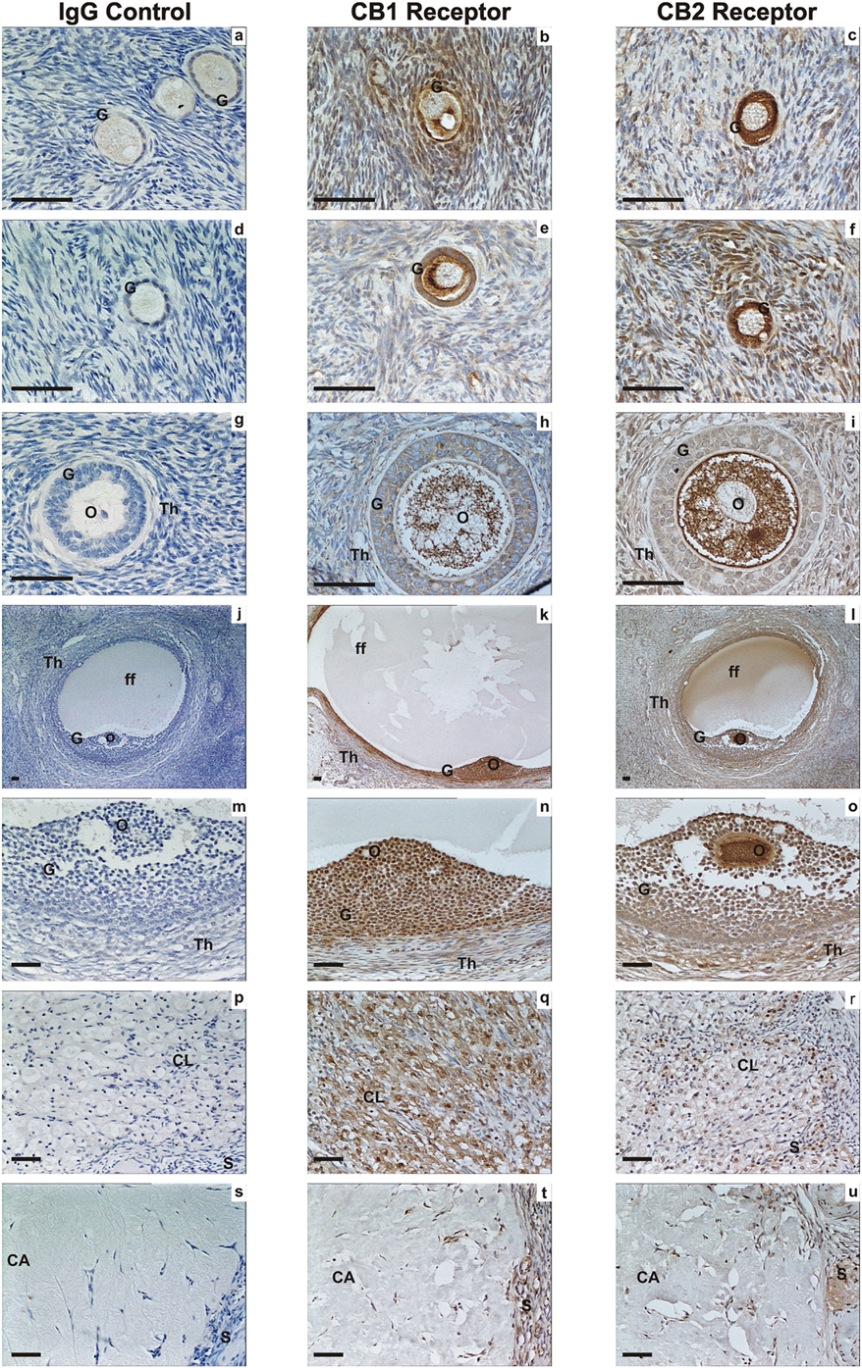
Immunohistochemical staining for CB1 and CB2 receptors. Images on the left side of the panel represent the negative control (IgG) and images in the middle are for CB1 on the right are for CB2. Images a, b and c are primordial follicle; d, e and f are primary follicles; g, h and i are secondary follicles; j, k and l are low power images of tertiary follicles; m, n an o are high power images of tertiary follicles, p, q and r are corpus luteum, r, s and t are images of the corpus albicans. Granulosa cells (G), theca cell layers (Th), the oocyte (O) and follicular fluid (ff) demonstrated CB1 and CB2 immunoreactivity as did lutein-granulosa cells of the corpus luteum (CL) and corpus albicans (CL), and septa (S) between lobes of these postovulatory bodies. The images are representatives from at least two structures and taken at 50×, 200× or 400× magnification. Bar = 50 µm. Ovarian follicles were classified as primordial, primary, secondary, tertiary, corpus luteum, and corpus albicans [43].

**Figure 3:**
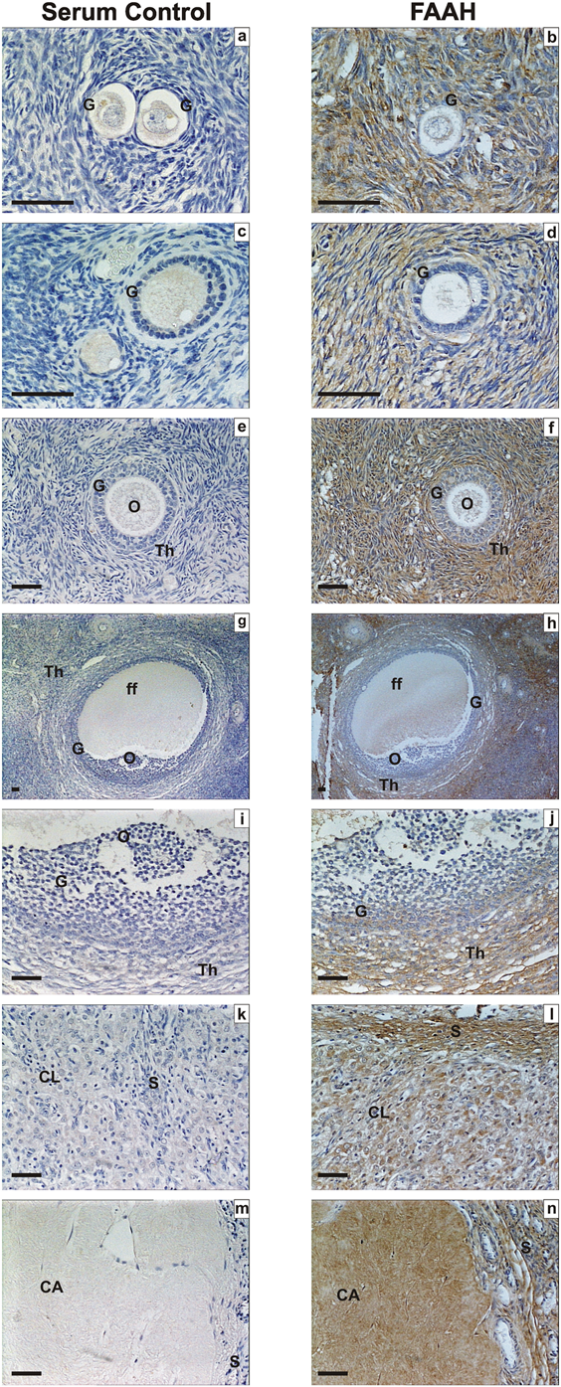
Immunohistochemical staining for FAAH. Images on the left side of the panel represent the negative control (non-immune rabbit serum and images on the right are for FAAH. Images a and b are primordial follicle; c and d are primary follicles; e and f are secondary follicles; g and h are low power images of tertiary follicles; i and j are high power images of tertiary follicles, k and l are corpus luteum, and m and n are images of the corpus albicans. Granulosa cells (G), theca cell layers (Th), the oocyte (O) and follicular fluid (ff) demonstrated FAAH immunoreactivity as did lutein-granulosa cells of the corpus luteum (CL) and corpus albicans (CL), and septa (S). There was a gradation of staining in the granulosa cells of the tertiary follicle with greatest intensity in the mural cells (panel j). The images are representatives from at least two structures and taken at 50×, 200× or 400× magnification. Bar = 50 µm.

**Figure 4:**
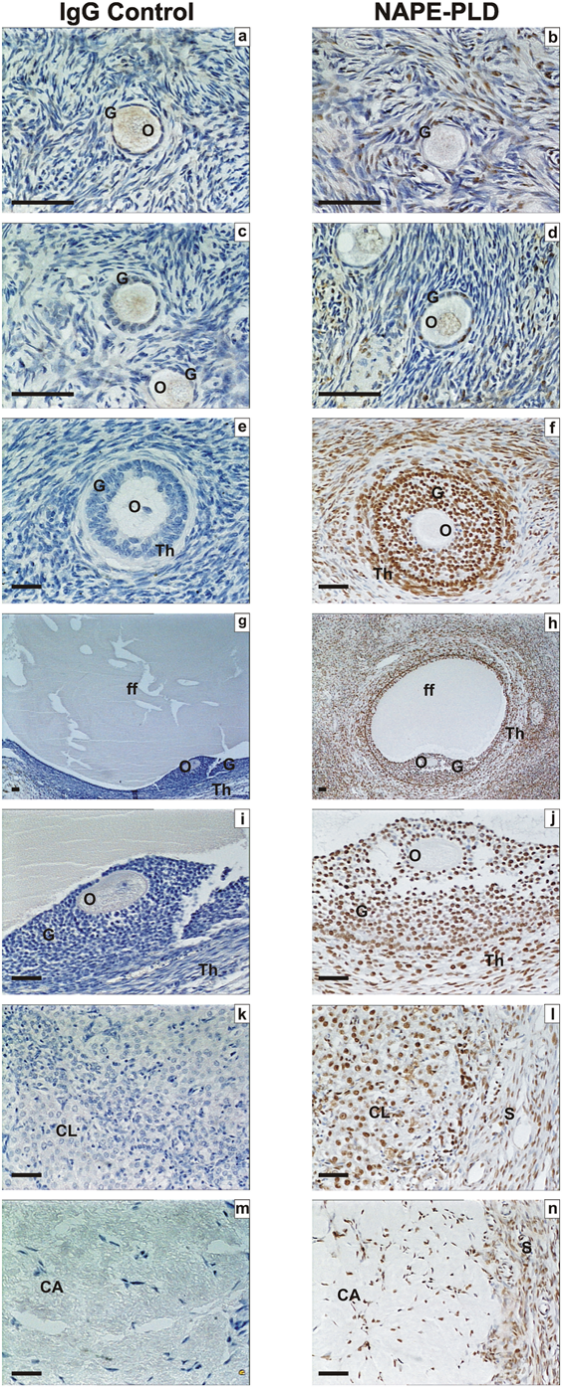
Immunohistochemical staining for NAPE-PLD. Images in the left side of the panel represent the negative control (rabbit IgG) and images on the right are for NAPE-PLD. Images a, and b are primordial follicles, c and d are primary follicles, e and f are secondary follicles; g and h are low power images of tertiary follicles, i and j are high power images of tertiary follicles, k and i are images of corpus luteum, and m and n are images of corpus albicans. Granulosa cells (G), theca cell layers (Th), the oocyte (O) and follicular fluid (ff) demonstrated NAPE-PLD immunoreactivity as did lutein-granulosa cells of the corpus luteum (CL) and corpus albicans (CL) and septa (S). The images are representative from at least two structures and taken at 50×, 200×, or 400× magnification. Bar = 50 µm.

Specificities for each of the antibodies used in these studies using control tissues are shown in Figure 1, using term fetal membranes for CB1, CB2 and FAAH and secretory phase endometrium for NAPE-PLD. Immunohistochemical analysis of the ovarian tissues for immunoreactive CB1, CB2, FAAH and NAPE-PLD revealed a widespread pattern of immunostaining in the ovarian cortex and medulla (Figures 2, 3, 4, and Table 1). The immunoreactivity for CB1 was observed in the medulla and the cortex. With regard to the follicles CB1 was localised in primordial, primary, secondary and tertiary follicles but mainly in the granulosa and theca cells of the secondary and tertiary follicles. Immunostaining was observed in the oocytes of these follicles and increased in the granulosa cells of the tertiary follicle and was observed in the corpora lutea and corpora albicantes (Figure 2). Likewise, CB2 (Figure 2o) was expressed in a similar manner as CB1 (Figure 2n) except the CB2 staining was more intense in the ovarian follicles in comparison to CB1 and more intense in the oocytes and granulosa cells in comparison to the theca cells (Figure 2c, 2f, 2i and 2o). However, FAAH immunostaining was not observed in the granulosa cells or the oocytes of primordial, primary, secondary or tertiary follicles but was expressed in the theca cells of secondary and tertiary follicles, and luteinized granulosa cells of the corpus luteum and corpus albicans (Figure 3l and 3n). NAPE-PLD immunostaining was occasionally observed in the oocytes of primordial follicles, but not in the granulosa cells of primordial or primary follicles (Figure 4). NAPE-PLD immunostaining was also absent from the oocytes of other types of follicles. However, NAPE-PLD was clearly expressed in the granulosa and theca cells of the secondary and tertiary follicles and corpus luteum, corpus albicans, and medulla (Figure 4). The intensity of immunostaining of NAPE-PLD in the corpus albicans (Figure 4n) appeared to be of a lower intensity when compared to that of FAAH staining in corpus albicans (Figure 3n).

**Table 1:** Immunohistochemistry localisation of the various components of the endocannabinoid system in the human ovary.

*Structure*	*Cell type*	*CB1*	*CB2*	*FAAH*	*NAPE-PLD*
Primordial Follicle	oocyte	+	++	−	−/+
	granulosa	+	++	−	+
Primary Follicle	oocyte	+	++	−	−
	granulosa	+	+	−	−
Secondary Follicle	oocyte	+	++	−	−
	granulosa	+	+	+	++
	theca	+	+	++	++
Tertiary Follicle	oocyte	−	++	−	−
	granulosa	++	+	−/+	−/++
	theca	+	+	++	++
Corpus luteum		++	+	+	++
Corpus albicans		+	+	−	+

Scoring system (−) = staining absent; (+) = staining visible; (++) = strong staining.

### IVF/ICSI-ET patients and follicular AEA measurements

A total of 37 women undergoing IVF/ICSI-ET were recruited in to this part of the study, their mean age and BMI were 33.28±5.08 years, 24.25±2.68 Kg/m^2^, respectively. Tables 2 and 3 show the demographic characteristics of these women and the medications used for controlled ovarian hyperstimulation.

**Table 2:** Characteristics of the 37 volunteers undergoing IVF/ICSI-ET.

*Category*	*Sub-Category*	*Mean/ number*	*SD*	*Range*
Age (years)		33.28	5.08	23–42
BMI (Kg/m^2^)		24.25	2.68	19.5–30
Infertility period (years)		3.86	2.45	1–14
Type of infertility:	Primary	26		
	Secondary	11		
Infertility cause				
	Male factor	16		
	Unexplained	11		
	Tubal	7		
	Endometriosis	1		
	PCOD+male	1		
	tubal+male	1		
Treatment:	ICSI	20		
	IVF	15		
	ICSI+IVF	2		
Basal hormones:				
	Basal FSH (IU/l)	5.69	1.68	2.5–9.9
	Basal LH (IU/l)	4.81	1.72	2–10

SD = standard deviation; Range = minimum to maximum values; BMI = body mass index; PCOD = polycystic ovarian syndrome; IVF = in-vitro fertilization; ICSI = intracytoplasmic sperm injection; FSH = follicle stimulating hormone; LH = luteinizing hormone.

**Table 3:** Details of the ovulation stimulation regimen, number of oocytes collected per cycle and number of embryos on the day of embryo transfer.

	*Mean/ number*	*SD*	*Median*	*Range*
Duration of stimulation (days)	11.55	1.38	12	9–15
Dose of Puregon (rFSH) IU/L	2251.08	1478.99	2025	675–6450
Dose of Menopur (hMG) IUL	3967.50	1895.86	3600	1950–7650
Dose of Puregon IU/L+Menopur IU/L	3425+1850	1242.22+482.18	3300+1650	2250–5725+1500–2400
Number of women stimulated by Puregon (rFSH)	25			
Number of women stimulated by Menopur (hMG)	9			
Number of women stimulated by hMG+rFSH	3			
Number of follicles aspirated per woman	16.41	6.89	15	2–29
Number of oocytes collected per woman	11.37	5.07	11	1–23
Total number of embryos per woman on the day of embryo transfer	5.30	3.09	5.50	1–14
Number of good quality embryos on the day of transfer	4.08	3.24	4	1–14

hMG = human menopausal gonadotrophins; rFSH = recombinant follicle stimulating hormone.

**Figure 5:**
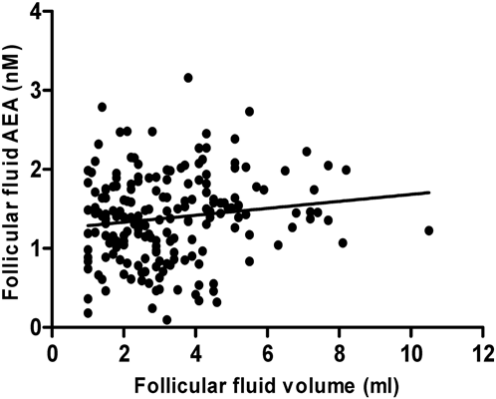
Spearman correlation between follicular fluid AEA concentration and follicle size. A positive correlation between the size of preovulatory follicle (in ml) and the concentrations of follicular fluids AEA from IVF/ICSI patients is shown. Follicular fluids from 193 follicles were analysed, R = 0.1304; P = 0.03.

A total of 260 follicles from these women were studied, of which 193 contained at least 1 ml of follicular fluid; their volume having been accurately measured. There was a significant (P = 0.035) positive correlation between follicular size and follicular fluid AEA concentration (Figure 5). The measured AEA concentration (1.43±0.04 nM) in the follicular fluid of the 172 follicles from which eggs were retrieved was significantly higher than that (1.26±0.06 nM) in the follicular fluid of 88 follicles where oocytes were not retrieved (P = 0.014).

**Figure 6:**
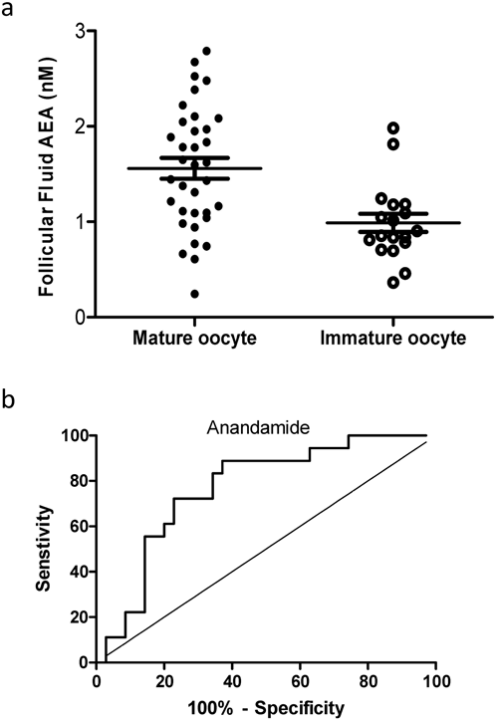
Prediction of oocyte maturity from follicular fluid AEA measurements. Panel a shows the follicular fluid AEA concentrations from follicles that gave rise to mature and immature oocytes during ICSI cycles. The long horizontal bar represents the mean and the shorter bars the sem. Panel b shows a receiver-operating characteristic curve (ROC) analysis for the prediction of the production of mature oocytes from follicular AEA concentration. The sensitivity and specificity relationship for measurements of anandamide in follicular fluid is plotted. The optimum cut off point for the identification of mature oocytes at 1.09 nM, provided a sensitivity of 72.2%, (95CI = 46.52% to 90.31%) and a specificity of 77.14% (95CI = 59.86 to 89.58%) and likelihood ratio of 3.16. The area under the curve was 0.768±0.067, (Mean±sem; 95%CI = 0.63–0.89, P = 0.001).

A total of 53 oocytes were retrieved from those undergoing ICSI and 35 of these were mature and 18 immature. The mean follicular fluid AEA concentrations (1.56±0.11 nM) in follicles with mature oocytes was significantly (P = 0.001) higher than that (0.99±0.09 nM) of the immature oocytes (Figure 6a). Furthermore, ROC curve analysis revealed that a cut-off level for follicular AEA concentrations that identified a mature oocyte, was 1.09 nM with an area under the curve of 0.76±0.07 (P = 0.001) with a sensitivity of 72.2% and a specificity of 77.14% (Figure 6b). After sperm injection and incubation, of the 35 mature oocytes, 21(60%) were fertilised; and 13 of these (61.9%) were good and 8 were poor quality embryos on the day of embryo transfer (48 to 72 hours later). The mean AEA concentrations in follicles that produced good (1.49±0.21 nM), and in those producing poor (1.48±0.19 nM) quality embryos were similar (P = 0.99).

There was no significant difference (P = 0.23) between the mean plasma AEA concentrations (1.52±0.07 nM) of the 37 women on the day of oocyte retrieval and the mean follicular fluid concentrations (1.42±0.05 nM) from all the follicles for each woman. In addition, the mean plasma AEA concentrations on the day of oocyte retrieval were similar (P = 0.27) to the values we previously reported in plasma AEA (1.33±0.16 nM) at the time of ovulation in natural cycles [34].

## Discussion

The endocannabinoid system in the human ovary has very rarely been investigated, and our recent observations that plasma anandamide concentrations increase at the time of ovulation in the natural menstrual cycle prompted us to look at the ovary in more detail [34]. Although Scheul *et al.* [21] quantified anandamide and some of its congeners in follicular fluid, there was sparse evidence in the literature that either confirmed this observation or explained the exact function of AEA in the human ovary. Therefore, our current study was designed to localise the endocannabinoid system in the human ovary and to examine if there was a role for AEA in relation to the physiologically vital processes that occur in the ovary, such as folliculogenesis, preovulatory follicle maturation or oocyte maturity.

To the best of our knowledge, this is the first time that the entire endocannabinoid system; i.e. the ligand, the receptors and the enzymes responsible for AEA regulation have been localised in the human ovary. The immunostaining showed widespread expression of CB1 and CB2 receptors in the medulla and cortex of the ovary. In the cortex the receptors were expressed in the granulosa cells of primordial, primary, secondary and tertiary follicles and in the theca cells of secondary and tertiary follicles. Immunostaining for both receptors was also observed in the corpus luteum and corpus albicans. In general, CB2 immunostaining was more intense than CB1 in the ovary, but interestingly, oocytes of follicles at all stages of development did not show positive expression of CB1 or CB2 except the oocytes of tertiary follicles, which expressed the CB2 receptor. These data suggest that the follicles and corpora are all likely to respond to AEA, but oocytes may not respond to AEA until the last stage of its development. These observations have led us to suggest that AEA may be involved in oocyte maturity, through the actions of the CB2 receptor. However, it is acknowledged that immunohistochemistry as the only approach investigating the endocannabinoid system in the ovary is limited, and that studies with other techniques, e. g. in vitro functional studies, if feasible, would be more comprehensive.

FAAH, the enzyme responsible for the degradation of AEA, was expressed only in theca cells of secondary and tertiary follicles, the corpus luteum and corpus albicans. These data suggest that AEA is acting in an autocrine manner on the granulosa cell to stimulate unknown phenotypic changes, and that an alternative degradation pathway that does not involve FAAH may be present in the granulosa cell. Indeed, recent evidence suggests that AEA can be converted to a prostaglandin E2-ethanolamine through the actions of cyclooxygenase 2, an enzyme that is expressed in the ovarian granulosa cell [35] and which is under leptin control.

NAPE-PLD on the other hand was expressed in the granulosa and theca cells of secondary, and tertiary follicles, the corpus luteum and corpus albicans suggesting that AEA is mainly produced from the granulosa of growing (secondary, tertiary) follicles but not from oocytes. Since NAPE-PLD was immunolocalised to the corpus luteum, and to a lesser extent the corpus albicans, this also suggests that AEA is synthesised by these from the two sources during the latter stages of the menstrual cycle.

These observations suggest that AEA probably acts mostly on CB2 receptors to produce its physiological actions in the ovary and that it is degraded in the theca cells and corpora lutea and albicantes. They also provide evidence that AEA is produced in the human ovary and that this process is probably under hormonal control as it is produced by growing follicles, the corpus luteum and corpus albicans. The evidence thus suggests that AEA affects the antral phase of folliculogenesis [36]. Nevertheless, recent evidence suggests that in the mouse, the main actions of endocannabinoids are mediated through the actions of the CB1 receptor causing an imbalance between E2 and P4 signalling [37]. The data presented herein are not at odds with this observation as Wang et al., did not examine the presence of the CB2 receptor, although they demonstrated that CB2 was not involved through the use of a specific CB2 antagonist. These observations therefore highlight a differential between murine and human ovarian physiology, as has been previously observed with other markers of ovarian physiology [38] and to our mind suggest that to really understand human ovarian function the human not murine ovary needs to be studied in more detail. Our volunteers from whom ovarian tissue was obtained were approaching the end of their reproductive age and therefore it would be interesting to replicate these studies in younger women, to ensure that the effect of age on ovarian physiology did not influence our observations.

The ovarian stimulation protocol used for IVF/ICSI women causes the development of several follicles with a wide range of sizes and at different developmental stages. The observed increase in follicular fluid AEA concentrations with increased follicle size and lower AEA concentrations in follicles from which oocytes were not retrieved indicate that AEA is probably involved in the maturation of follicles or the oocyte. Since oocyte maturity is currently assessed subjectively by embryologists there is a need for a more objective method of assessment. Although many potential biomarkers (estradiol and testosterone (39), inhibin B [40], BMP-15 [41]) in follicular fluid have been suggested to discriminate between mature and immature oocytes, none of these are in current clinical practice. In this regard, the ROC analysis demonstrating that a 1.09 nM follicular fluid AEA level was predictive of mature oocytes in 77.14% of the cases is especially encouraging. Although this study clearly indicates that follicular fluid AEA levels are associated with both follicle and oocyte maturity, there are likely to be a number of other factors that influence oocyte maturity and quality in the local hormonal environment that we do not know about [42]. It would be ideal to relate the follicular fluid and plasma AEA levels with oocyte/embryo quality in women with different causes of infertility; however, the numbers in this study were too small for such sub-analyses. We believe that follicular fluid AEA levels should be further investigated as a possible biomarker for the assessment of oocyte maturity in advanced reproductive technology procedures. This needs to be done in conjunction with other biomarkers.

With regards to the embryo quality we did not find a significant association between follicular fluid AEA concentrations and embryo quality. This suggests that several other factors including sperm quality are involved in determining embryo quality.

The fact that there was no significant difference between plasma and follicular fluid AEA concentrations at the time of oocyte collection suggests that systemic AEA concentrations reflect ovarian AEA levels. Also, plasma AEA concentrations in women undergoing IVF/ICSI at the time of oocyte collection were similar to those found in women at ovulation in natural cycles [34] which suggest that plasma AEA is involved in the ovulation process whether it occurred naturally or was stimulated.

### Conclusion

This study is a step forward in helping with the understanding of the mechanisms of action of AEA in the human ovary. A better understanding of the mechanism whereby AEA is produced in the human ovary and how it affects oocyte maturity, whether nuclear or cytoplasmic maturation, may help in the development of strategies which may improve the outcome of ART. Furthermore, there is a need to undertake larger studies to investigate the potential use of follicular fluid AEA measurement as a predictor of oocyte maturity.
